# Aspects of family caregiving as addressed in planned discussions between nurses, patients with chronic diseases and family caregivers: a qualitative content analysis

**DOI:** 10.1186/s12912-017-0231-5

**Published:** 2017-07-11

**Authors:** E. I. Hagedoorn, W. Paans, T. Jaarsma, J. C. Keers, C. van der Schans, M. Louise Luttik

**Affiliations:** 1 Hanze University of Applied Sciences, Research Group Healthy Ageing, Allied Health Care and Nursing, Groningen, The Netherlands; 2 University of Groningen, University Medical Center, Department of Health Psychology, Groningen, The Netherlands; 3 Hanze University of Applied Sciences, Research Group Nursing Diagnostics, Groningen, The Netherlands; 4 Linköping University, Department of Social and Welfare Studies (ISV), Linköping, Sweden; 5 Martini Hospital, Groningen, The Netherlands; 6 University of Groningen, University Medical Center Department of Rehabilitation Medicine, Groningen, The Netherlands

**Keywords:** Family caregiving, Nursing processes, Elderly, Chronic diseases, Hospitalization

## Abstract

**Background:**

Caregiving by family members of elderly with chronic conditions is currently intensifying in the context of an aging population and health care reform in the Netherlands. It is essential that nurses have attention for supporting roles of family caregivers of older patients and address family caregiving aspects on behalf of the continuity of care. This study aims to explore what aspects of family caregiving were addressed during planned discussions between nurses, patients and family caregivers in the hospital.

**Methods:**

Qualitative descriptive research was conducted using non-participant observation and audio-recordings of planned discussions between nurses, older patients and their family caregivers as they took place in the hospital. Through purposive sampling eligible patients (≥ 65 years) with one or more chronic conditions were included. These patients were admitted to the hospital for diagnostics or due to consequences of their chronic illness. Retrospective chart review was done to obtain patient characteristics. Data were collected in November/December 2013 and April/May 2014 in four hospitals. Qualitative content analysis was performed using the inductive approach in order to gain insight into addressed aspects of family caregiving.

**Results:**

A total of 62 patients (mean age (SD) 76 years (7.2), 52% male) were included in the study, resulting in 146 planned discussions (62 admission and discharge discussions and 22 family meetings). Three themes were identified regarding addressed aspects of family caregiving. Two themes referred to aspects addressing the patients’ social network, and included ‘social network structure’ and ‘social network support’. One theme referred to aspects addressing coordination of care issues involving family caregiving, referred to as ‘coordination of care’.

**Conclusions:**

During discussions nurses mostly addressed practical information on the patients’ social network structure. When specific family caregiving support was addressed, information was limited and nurses did not seem to explore the nature of the family support. Patients discharge and after care needs were addressed occasionally as aspects of coordination of care. Current nursing policies could be evaluated on nursing and family oriented theories. Implications for education could include mirroring study findings with nurses in a group discussion to enhance their awareness on family caregiving aspects.

**Electronic supplementary material:**

The online version of this article (doi:10.1186/s12912-017-0231-5) contains supplementary material, which is available to authorized users.

## Background

Extensive research shows a positive relationship between family caregivers’ support and optimization of self-care abilities of elderly individuals experiencing chronic diseases [1–5]. Family caregivers are expected to support their chronically ill family member more due to an increasing shift from professional to informal care [6]. In the light of these developments, it is essential that nurses have attention for support offered by family caregivers of older patients with chronic conditions who are admitted to the hospital. Therefore, nurses need to address family caregiving aspects in their nursing practice on behalf of the continuity of care of the patient in the hospital. Too little attention for supporting roles of family caregivers during the hospitalization of their ill relative could lead to discontinuity of care after discharge.

The importance of family caregiver support of chronically ill elderly is a relatively new concept in the Netherlands which is receiving more attention the last ten years [7, 8]. Especially in primary care, many initiatives have arisen to support family caregivers in the care of older people with chronic conditions at home [1, 3, 5, 9–11]. However, recognizing that these issues are also important in hospitals in the Netherlands is relatively new. In the Netherlands, outcome indicators such as patient health (mobility; mortality; health status) and costs related to family care outcome indicators are not yet explored and can be seen as one of the next steps for further research.

International studies show that family caregivers can play an active role as an intermediate between their chronically ill family member and health care professionals, especially nurses [12–15]. Li and colleagues [16] identified three major roles of family caregivers: providing care to the patient, working together with the health care team, and taking care of themselves. Nurses need to view these family caregivers as partners of care with the health care team in order to accommodate in their roles [16].

In western societies, governmental policies are increasingly shifting care from professional health care to informal care expecting family caregivers to assume the caregiving role for their chronically ill family member. Until recently, the Netherlands had a welfare society like many other European countries, and home care support for elderly discharged out of the hospital was a matter of course. Now the Dutch government has implemented a ‘participatory society’, meaning that citizens are expected to take on more responsibilities by activating their own informal network [6]. Family caregivers are expected to take on more caring responsibilities after their chronically ill relative is discharged out of the hospital because home care support is not always provided.

Chronic diseases, such as congestive heart failure, generally have a significant impact on the quality of life of older adults and their family caregivers [3, 11]. These older adults and their family caregivers usually face a lifelong challenge with medical treatments and required life style changes which are summarized as self-care [17]. Elderly experiencing chronic diseases are more frequently hospitalized for diagnostics or due to the consequences of their chronic illness [18]. In general, the length of hospital stay is shortening, and as a result these older patients may not achieve a secure health status before being discharged. The care and support that family caregivers offer their ill family member post-discharge becomes more complex often resulting in greater demands on family caregivers [19]. Therefore, it seems essential that nurses identify possible family support of older patients with chronic diseases and determine to which extent family caregivers should be involved as partners in care.

It is evident that nurses play an important role in addressing family caregiving aspects during hospitalization of their chronically ill relative [19–21]. According to family nursing theory, primary goals of interactions between nurses and patients are described as gathering relevant information about the patient and their family [22]. Assessment of the elderly patients’ family caregiving network can help nurses understand and optimize the support of family caregivers during hospitalization and in the transition back home. Nurses should collaborate with the patient and family caregivers in order to maintain continuity of care as part of the professional profile, and subsequently, promoting the patient’s self-care [23].

Besides daily patient contact, there are a number of planned discussions between nurses and patients that can be considered elements of a structured nursing process, like admission and discharge discussions and family meetings [23]. Research shows that planned discussions, like admission and discharge discussions and family meetings could offer nurses opportunities to address family caregiving support on behalf of the continuity of care of their ill family member [20, 24]. Still, little is known on what aspects of family caregiving are addressed during planned discussions as part of the nursing process. Aspects can be seen as relevant issues regarding supporting roles by family caregivers of elderly patients with chronic diseases.

Thus far, research related to family caregiving has focused on perceptions of nurses, patients and family caregivers about their mutual interactions and involvement. This research has generally been compiled from discussions [13, 14, 21, 25–29], focus groups [15, 30] participant observations [31], or surveys [20, 32–34]. Research on perceptions and opinions aims to gain insight into how individuals perceive and experience certain situations. Observations and audio-recordings of actual planned discussions as part of the everyday business can be used to capture the social setting in which people function. It can ascertain whether what people actually say they do and what they genuinely do, correspond [35]. Therefore, observations and audio-recordings of planned discussions can provide an opportunity to study how opinions and perceptions correspond or differ from situations in reality. More insight into what aspects of family caregiving are actually addressed during planned discussions can offer starting points to enhance involvement of family caregivers as partner in care when desirable.

## Methods

A qualitative descriptive design was used in order to gain insight into what aspects of family caregiving were addressed during planned discussions between nurses, older patients and, if present, their family caregivers in the hospital. Non-participant observations and audio-recordings of planned discussions were employed in order to provide accurate information on what aspects of family caregiving were addressed. This method allowed researchers to be present during discussions without participating, to observe, listen, and take field notes more freely [35]. The purpose of taking field notes was to write down relevant aspects that could not be heard on the audio-recordings, including who were present during planned discussions, and the duration of discussions. Retrospective chart review was employed to obtain patient characteristics, specifically, age, living status and number and type of chronic diseases.

### Participants

A purposive sampling approach was employed to include patients 65 years or older who were experiencing one or more chronic diseases and were admitted to the hospital for diagnostics or due to their chronic illnesses. Examples of admission reasons were nausea, pneumonia, Chronic Obstructive Pulmonary Disease exacerbation and fluid retention**.** Patients were excluded if they were living in a care facility or had been admitted for day treatment. Nurses who conducted planned discussions with enrolled patients were included as participants in the study. Nurses had an Associate Degree or Bachelor Degree in Nursing. Nurses in the Netherlands who perform daily nursing tasks are also responsible for conducting admissions and discharge discussions, and family meetings with patients admitted to the hospital. Family caregivers of enrolled patients were included as participants when they were present during planned discussions between patients and nurses.

#### Planned discussions

Planned discussions are planned meetings between nurses and patients as part of the nursing process and refer to admission and discharge discussions and family meetings as they take place as part of the usual care on the hospital ward. The nature of the admission interview is structured. Nurses perform an admission interview soon after or at least within 24 h of admission. The admission forms used by nurses in participating hospitals were based on the functional health patterns of Gordon [36]; a format with eleven pre-structured domains: Health perception; Nutritional – Metabolic; Elimination; Activity – Exercise; Cognitive – Perceptual; Sleep – Rest; Self-perception – Self-concept; Role – Relationship; Sexuality – Reproductive; Coping – Stress Tolerance; Value – Belief Pattern. Family meetings referred to a planned meeting with patients, their family caregivers and professionals (nurses and physicians). These meetings did not take place structurally on all hospital wards, but when they were planned, family caregivers were invited to be present. The nature of this meeting is that of an informational meeting. Discharge discussions referred to planned meetings between a nurse and patient just before the patient is discharged home. The dynamic of the family meeting and discharge interview is an open discussion approach with a balance between open and closed ended questions with the aim to inform and to be informed.

### Data collection

Data were collected in two 4-weeks sessions in November–December 2013 and April – May 2014. Four general hospitals in the northern part of the Netherlands participated, with a total of 13 hospital wards aimed at elderly patients experiencing chronic conditions. Patients admitted to these hospital wards were not in acute life-threatening situations.

Data were collected by fourth year bachelor nursing students who received a 24-h training course specifically designed for this study. This course consisted of instructions on how to take adequate field notes and collect patient information from patient’s charts. Using simulations, data collectors practiced how to approach patients and perform non-participant observations. Two data-collectors were assigned to each hospital ward. Every day, nurses in charge screened new admitted patients for possible participation in the study. When eligible, patients were identified and data- collectors were notified of the time of the admission interview. Data-collectors approached the patient for participation in the study and observed and audio-recorded all planned discussions between nurses, patients and family caregivers as they took place on the hospital ward. During the observation, data collectors made notes of the duration time of the planned discussions, and who were present. Retrospectively, patient characteristics were obtained by chart review.

### Ethical considerations

Ethical approval was obtained from the Research and Ethical Committees of the Medical Center Leeuwarden, The Netherlands (nWMO42/2014). Participants received written and oral information about the study. Written informed consent for the study was obtained from the patients, including consent for publication. Nurses were informed about the study and asked to participate by the hospital managers. In order to minimize socially desirable behavior, nurses were told that the aim of the study was to gain insight into what aspects were addressed during planned discussions in general. Also, data-collectors visited participating wards prior to data collection to become ‘familiar faces’ to nurses [35]. Family caregivers were asked verbal permission at the time of the planned discussion. Nurses, patients and family caregivers were allowed to refuse participation. Data were transcribed anonymously.

### Data analysis

Qualitative content analysis was performed using the inductive approach as described by Elo and Kynga [37]. Content analysis is well-suited to analyze the multifaceted, important, and sensitive phenomena of nursing [37]. Audio-recordings of all discussions were transcribed and anonymized into verbatim transcriptions. Each discussion was transcribed in Dutch and analyzed using the qualitative data analysis program Atlas ti^®^ [38]. The first author and a research assistant initially analyzed transcriptions separately. Sections of texts regarding aspects of family caregiving were identified and described into ‘codes’. Codes were critically examined and overlapping codes were further refined and reduced in number by grouping them together into sub-categories and categories [37]. Based on discussion, consensus was reached resulting in a joint category list. Data were subsequently read again in order to assure that subcategories and categories were complete. In addition, the category list was discussed by the first and second author using the original data as a reference and categories were conceptualized into themes. Identified subcategories, categories, themes, and quotes were translated into English by a translator for publication purposes.

To increase dependability the method of research and results were discussed by all authors. Planned discussions were audio-taped and observed as they took place, as part of the everyday business on the hospital wards [39]. This allowed us to gain insight into aspects being addressed as they acutally occur as part of the nursing proces. To improve credibility, peer review was used to validate data analysis by the second and sixth author [40]. In case of disagreement consensus was reached by discussion.

## Results

Initially, planned discussions of 88 patients were included. In 62 (70%) of these patients, an admission as well as a discharge discussion was audio recorded. In total, planned discussions of 62 patients were employed for data analyses. Due to primarily logistic reasons a number of planned discussions (*n* = 26) were not audio-taped, e.g., early discharge, change of ward, or because the patient had died. Of the 62 included patients, 18 patients were from neurology, 17 from pulmonary, 15 from internal medicine, 10 from cardiology, and two from geriatrics. A total of 146 audio-taped planned discussions of included patients were used for data analysis, of which 22 family meetings. Based on data analysis of transcriptions it seemed to be clear that no new codes and themes were generated and that data saturation was reached.

### Patient characteristics

Mean (SD) age of patients was 76 years (7.2), ranging from 65 to 94 years. A total of 36 (58%) patients were experiencing two or more chronic diseases (Table 1). Mean (SD) length of stay was eight days (5.4), ranging from two to 29 days, with 24 (39%) patients staying ≤5 days, 23 (37%) patients stayed six to ten days, and 15 (24%) patients stayed ≥10 days.

**Table 1:** Patient demographics

Demographics		Number	Percent
Gender	Male	32	52
	Female	30	48
Living status	With partner	39	63
	Living alone	20	32
	Living with child	2	3
	Living in a social community	1	2
Number of chronic conditions	1 chronic disease	20	32
	2 chronic diseases	20	32
	3 ≥ or more chronic diseases	22	36
Type of chronic conditions^a^	Cardiovascular disease	32	52
	Pulmonary disease	24	39
	Diabetes	20	32
	Blood pressure issues	13	21
	Stroke	9	14
	Rheumatism	9	14
	Kidney disease	4	6
	Hypercholesterolemia	4	6
	Parkinson’s	3	5
	Thyroid problems	3	5
	Cancer	3	5
	Other^b^	5	8

^a^Total is >100% due to more than one chronic condition per patient

^b^Anemia, bowel disorder (2), esophageal disorder, lymph disorder

In order to give the reader an impression of the context in which these planned discussions took place, a short overview is given of the duration of the discussions, and who were present. The mean (SD) duration of admission discussions was 21 (8.3) minutes, that of family meetings was 17 (7.1) minutes, and that of discharge discussions 5 (4.7) minutes. Of the 22 planned family meetings, 7 (32%) meetings were held on one hospital ward where family meetings were structurally planned. Field notes taken during observations on the presence of family caregivers indicate that in 107 (73%) of the planned discussions one or more family caregivers were present. During admission discussions family caregivers were present 47 times (76%), during family meetings 18 (82%) times and during discharge discussions 42 (68%) times. Mostly partners or children, or a combination of partners and children of patients were present during the planned discussions. Additional file 1 gives an overview of all the participating patients and presence of their family caregivers.

#### Aspects of family caregiving as addressed in planned discussions

Based on the analysis of the planned discussions, three themes were identified regarding aspects of family caregiving. Two themes referred to aspects addressing the patients’ social network, and included ‘social network structure’ and ‘social network support’. One theme referred to aspects addressing coordination of care issues involving family caregiving, referred to as ‘coordination of care’. An overview of addressed aspects of family caregiving is given in Table 2. In describing the frequency in which categories and themes were addressed the term ‘a few times’ was used; referring to two to three times.

**Table 2:** Aspects of family caregiving

Themes *Categories* Subcategories	
Social network structure	
*Contact information of family members*	
− Contact information of primary contact persons	
*Family composition*	
− Living status	
− Number of children	
*Social network availability*	
− Social network available	
Social network support	
*Support by family caregivers*	
− Support by family caregivers at home	
− Support by family caregivers in the hospital	
*Family caregiver role*	
− Patient is family caregiver at home	
− Family caregiver burden	
Coordination of care	
*Aspects related to patient hospital stay*	
− Hospital ward information for family caregivers	
− Coordination of family meeting	
*Aspects related to patient discharge*	
− Coordination of discharge date	
− Discussing after care at home	
− Life style instructions	
− Review home medication	
− Contact information on follow-up care	
− Contact information in case of worsening symptoms	

##### Social network structure

The theme ‘social network structure’ concerned practical information on patients’ social network and consisted of three categories, contact information of primary contact persons, family composition, and social network availability. During admission discussions nurses generally asked practical information about the patients’ social network, including patients’ primary contact person(s), their phone numbers, patients’ living status, and the number of children. This information was often addressed by asking a series of closed ended questions, as illustrated in the following quotes:

Nurse: Let’s see, are you married? Patient: Yes Nurse: …. Do you have children? Patient: Yes. Nurse: Yes? – and all out of the house? Patient: out of the house, yes. Nurse: And how many children do you have? Patient: One. Nurse: One? A son or daughter? Patient: Daughter. [Patient 7 hospital A, pulmonary unit, admission]*.*

Nurse: How many children are.. do you have? Spouse: ..a son and a daughter. Nurse: okay. And do you live nearby (nurse asks the son and daughter)? Son: Oh yeh. Nurse: I ask that in case a meeting needs to be held.. that you .. eh.. that we should not think : oh they must come from far. Daughter: No (mentions her residence) so that’s not far [Patient 15 hospital B neurology unit, admission]

Nurses occasionally addressed the quality of contact between patients and their children during the admission discussion, as exhibited in the following quote:

Nurse: May I ask, do you have good contact with each other? Daughter and son both answer: Yes. Nurse: Yes, …normal contact? Daughter: Well, yes. Nurse asks patient: Are you in touch with all your children? Son and daughter both answer: Yes. Nurse: Well, then, I'll just write that down. Is in touch with all the children [Daughter and son of patient 10, hospital B, neurology unit, admission]*.*

Nurses also occasionally addressed the availability of the patients’ social network during admission conversations as demonstrated in the following quotes:

Nurse: Who can you turn to after discharge? Patient: To my wife. Nurse: Yes? Patient: my children all live far away (patient mentions where the children live) [patient 4, hospital B, cardiology, admission]*.*

##### Social network support

The theme social network support referred to aspects related to support provided by patients’ social network, and consisted of two categories, *support provided by family caregivers’* and *family caregivers’ roles*. Subcategories identified were support offered by family caregivers at home and support by family caregivers in the hospital. The subcategory ‘support of family caregivers offered at home’ referred to specific support with activities of daily living, as in housekeeping tasks, transportation to the hospital and assisting with or monitoring medication intake, etc. This aspect was mostly addressed on the initiative of patients themselves or by their family caregivers. When informal support by family caregivers was addressed it concerned mostly information on the presence of support, as illustrated by the following quotes:

Nurse: And, do you need help with personal care? Spouse: Yes. Patient: Sure enough. Nurse: Do you have homecare? Spouse: Yes, I am the homecare. Nurse: You are the homecare? Spouse: Yes. Nurse: Yes, also of .. Spouse: Yes [Patient 2, hospital D, pulmonary unit, admission]*.*

Nurse: And, at home, do you still do everything yourself ? Patient: Yes, .. I have help. Nurse: Okay, home care? Patient: No, just regular help on Fridays Nurse: Yes, for in the household? Patient: Yes, but nothing else. My help is.. sits there (she looks at her husband) Nurse: Yes (they laugh) Spouse: And with pleasure. Nurse: that’s good (she is typing) [Patient 3, Hospital B, cardiology unit, admission]

A few times did nurses elaborate on possible support of family caregivers for their partner at home during admission discussions, as illustrated in the following quote:

Nurse: no further help? Spouse: No, he does it all himself. Nurse: You help Mr.? , or not? Spouse: No. Patient: well, ..you do the stockings. Spouse: the stockings that's all. Nurse: Okay, .. Spouse: He can bathe and dress himself. Nurse: Yes. Patient: Yes, I can dress myself, but.. Nurse: bathing also? Patient: Yes Nurse: Even now, when you're so short of breath? Patient: Yes, yes this morning too, …. Yes, but don’t asks me how long it takes me [Patient 1 and Spouse, hospital A, pulmonary unit, admission]*.*

The subcategory ‘support of family caregivers in the hospital’ was addressed twice, once by a nurse and once by a family caregiver. In both cases it concerned family members being present in order to keep patients calm at night, as illustrated in the following quote:

Nurse: Can we call you when ..if it becomes difficult in terms of unrest? If we feel that we cannot keep him in his bed?

Son: Yes, of course

Nurse: It’s not always possible, that’s why I asked

Son: yes [Patient 7 and Son, hospital C, neurology unit, admission]

The category *family caregiver role* concerned specific aspects about the nature and impact of support given by family caregivers at home. Two subcategories were identified: ‘patient is family caregiver at home’ and ‘family caregiver burden’. In two admission discussions the specific role of the caregiver was addressed by a patient and a family caregiver, when it was obvious that these admitted patients were family caregivers themselves. A daughter addressed this issue at the end of an admission interview:

You haven't asked about the home situation. My father, …he suffered a stroke some years ago and had heart surgery two years later. So, now, he is home alone, but the fact is that my mother, well, she now has to take full care of him as well, you see. She helps him shower and, well, cooks him dinner and things like that. ...but that would perhaps be something important to mention, wouldn't it?” Nurse: I'll certainly write that down, yes, of course [Daughter of patient 13, hospital C, Internal medicine, admission]*.*

The subcategory of ‘family caregivers’ burden’ referred to the ability of family caregivers to deal with the care and support offered to their chronically ill partner or family members at home. A few times did nurses address family caregivers’ feelings of burden when the family caregivers’ burden was evidenced, as illustrated by the following quote:

Nurse: and you still manage, the care, is it enough? Spouse: Yes...that, I can get more Nurse: oh, that is possible? Spouse: yes he is already so far, he can be admitted to (name nursing home), but I don’t want to. Nurse: No, okaySpouse: I want to keep him home as long as I can [Spouse of patient 3, hospital D, Pulmonology, admission]*.*

#### Coordination of care

The theme coordination of care referred to aspects addressing coordination of care issues related to family caregiving, and included the categories ‘aspects related to the patients’ hospital stay’ and ‘aspects related to the patient discharge’.

*Aspects related to hospital stay* consisted of two subcategories; hospital ward information and coordination of family meetings. ‘Hospital ward information’ referred to information generally given to family caregivers regarding hospital ward information, such as visiting hours and contact phone numbers. Nurses structurally handed over this information to patients, or if present, family caregivers during admission discussions. The subcategory ‘coordination of family meetings’ referred to nurses’ initiatives to coordinate a family meeting in consultation with patients or family caregivers. A few times did nurses specifically coordinate a date and time for a family meeting with patients and their family caregivers during a planned discussion. On one hospital ward, family meetings were structurally planned with patients and family caregivers.

The category *aspects related to patient discharge* concerned specific actions or information regarding patients’ discharge and follow-up care on behalf of the continuity of care of family caregiving. Subcategories included ‘coordination of discharge date’, discussing after-care at home’, ‘instructions on life style and home medication’ and ‘contact information on follow-up care’ or ‘in the event of worsening symptoms’. Nurses rarely discussed a discharge date with patients or family caregivers during planned discussions. A few times did nurses address the patient home situation in order to coordinate possible after care activities, as illustrated in the next quote:

Nurse: Has everything been arranged at home regarding assistance...the necessary care? Patient: My partner is doing...well, we live together, so ... Nurse: Yes, you're taking care of things together Patient: Yes [Patient 11, hospital C, neurology unit, discharge]*.*

During discharge discussions nurses regularly reviewed the home medication list with patients and or family caregivers. Family caregivers occasionally asked specific questions about changes in the patients’ home medication, but mostly during family meetings.

Aspects of follow-up care were addressed occasionally during planned discussions. This mostly concerned contact information of health care professionals or organizations on follow-up care. A few times did nurses as well as family caregivers specially address aspects on who to contact in case of worsening symptoms of their ill family member.

## Discussion

This is the first study to describe what aspects of family caregiving are discussed during planned discussions as they took place between nurses, patients and, if present, their family caregivers in the hospital. Aspects of family caregiving that nurses most frequently addressed as part of these planned discussions were aspects on the structure of the patients’ social network as in collecting practical information about patients’ primary contact persons. Nurses occasionally addressed the availability of the patients’ social network although nurses did not explore possible family caregiving roles while addressing these aspects. Family caregivers’ support at home was also addressed occasionally and mostly by patients and family caregivers themselves. When specific support of family caregivers was addressed, information was limited and nurses did not further elaborate on this information to explore the nature of family support.

Nurses structurally handed over hospital ward information to family caregivers, such as visiting hours and contact phone numbers. During discharge discussions, nurses regularly reviewed home medications with patients and or family caregivers. Nurses occasionally addressed coordination of care aspects with family caregivers related to the patients’ discharge and after care, especially during family meetings and discharge discussions.

During the hospitalization of elderly patients experiencing chronic conditions, admission and discharge discussions were structurally held, although the mean duration of discharge discussions was short. Family meetings were held in only a third of the patients’ hospitalizations. Results also showed that family caregivers were actively involved and largely present during these planned discussions.

An explanation for a finding that nurses mostly collect practical information on patients’ social network might be that nurses do not see family caregivers as relevant for their nursing practice. This is in line with findings that show that nurses traditionally perceive the patient as their main responsibility [25, 41]. Also, studies indicate that nurses perceive family caregivers as relatives of patients who primarily require information or who have practical problems that need to be solved [24, 42]. This might also be an explanation why nurses rarely elaborated on aspects of family caregiving in order to explore specific support. It is likely to assume that hospital nurses do not see family caregiving support at home as a relevant aspect in their nursing care, even though the Dutch professional nursing standard states that it is expected from the nurse that there is cooperation with the care recipient and its family caregivers [43]. A finding that nurses rarely elaborate on specific support of family caregivers may also reflect that family nursing theories are not yet imbedded as a structural component of their nursing assessment. According to family nursing theory primary goals of interactions between nurses and patients should focus on gathering relevant information not only on the patient but also their family caregivers [23].

Another reason why nurses rarely elaborate on specific family caregiving support might be that they are unaware of implications of the recent policy changes regarding the changing role of family caregivers. Nurses might be aware that professional support after discharge out of the hospital is not a matter of course anymore. Still, it is likely to assume that nurses are unaware of the implications of the shift from formal to informal care for their own role as hospital nurse. Nursing staff who believe family presence is important are more likely to include families in daily care [33]. Still, it is also important for nurses to realize that family caregivers who are present in the hospital are not necessarily willing to participate in patients’ care.

Results also show that family caregivers play an active role during planned discussion, in asking questions and sharing information with nurses. This is in line with earlier studies showing that family caregivers play an active role as an intermediate between their ill relative and other health professionals [12, 16, 44].

Still, it seems that nurses and family caregivers experience communication difficulties due to lack of time, of opportunity and of skills on the part of nurses [25]. Findings of this study show that nurses seem to have sufficient opportunity and time during planned discussions to address aspects of family caregiving, especially during admission discussions and family meetings. Still, it is likely to assume that nurses may not have sufficient communication skills to talk to family caregivers. Hallgrimdottir and colleagues [34] also found that nurses might have insufficient skills in communicating with family caregivers. On the other hand, Lindhardt and collagues [45] found that nurses stated to have received sufficient education in communicating with family caregivers. Therefore, it is likely to assume that nurse’s perceptions can differ from what takes place in reality. Education in the principles of more family centered care might enhance nurses’ awareness of the role of family caregivers in regard to self-care abilities of older patients in the hospital. LeGrow and Rossen [46] found that based on an 8-h educational workshop presenting a family system nursing approach, the majority of nurse participants made a significant cognitive shift toward nursing the family. Wright and Leahy [22] suggest that when nurses ask three key questions they can better involve family members in family health care. Finally, another explanation for findings that nurses mostly collect practical information on patients’ social network might also be that nurses primarily adhere to the outline of the admission form, as, was also found by Jones [47]. Admission forms should be used as a directive for nurses to identify relevant patient issues in order to compose a plan of care. Despite family issues mentioned in Gordon’s functional health patterns [36], nurses do not seem to explore the underlying relevance of family caregiving related questions in order to identify possible family caregiving aspects.

### Strengths and limitations

Strengths of this study include extensive audio-recorded data and observations of planned discussions as part of the everyday business on hospital wards. Consequently, we were able to obtain a more realistic perspective of what was actually discussed during planned discussions regarding aspects of family caregiving. Subjectivity could possibly have been of influence in identifying the themes; however, we tried to minimize the chances because data were transcribed verbatim, and interpreted by three researchers. We also attempted to minimize the influence of subjectivity by applying the method of seeking consensus.

The presence of the data-collectors may have impacted nurses’ discussions, and therefore might have influenced results. Still, the impact was held to a minimum because the method of non-participant observation was used and data-collectors shadowed nurses for a number of days prior to the data-collection period in order to become a familiar face for nurses [35]. By using the purposive sampling method, we intended to include all eligible patients during the period of data collection. Still, it is plausible that a small number of eligible patients might not have been identified quickly enough for data collectors to be on time for the admission interview. Based on field notes, no other relevant aspects emerged which had influence on the analysis of the data. Other interactions between nurses, patients and family caregivers that took place outside of the planned discussions were not part of the study; therefore it is possible that other relevant information may have been missed.

## Conclusions

During planned discussions between nurses, patients, and family caregivers, nurses mostly collected practical information on the patients’ social network structure, like marital status and number of children. Nurses occasionally addressed the availability of the patients’ social network. When specific support of family caregivers was addressed the information was limited and nurses did not seem to elaborate on this information to explore the nature of the family support. This study is unique in its method in observing planned discussions between nurses, patient and (if present) their family caregivers as they actually occur in the hospital. Results show gaps in current nursing care related to aspects of family caregiving and provide starting points for improvements and further research.

### Implications

This study illustrates problem areas that should be acknowledged in the interest of international nursing practice. Current planned discussions seem to offer nurses opportunities to address aspects of family caregiving, however, the content and form require improvements. Implications for policies could be that current nursing policies, like admission forms, should be viewed in the light of family oriented theories. This could improve discussion on aspects of family caregiving, like the nature and intensity of family caregiver support at home. Also, current nursing policies should be evaluated in the light of the most recent professional standards. Planned discussions, especially family meetings, may offer nurses opportunities to communicate with patients and family caregivers on relevant aspects of family caregiving. Since family meetings were held structurally on only one of the hospital wards, we recommend to hospital administrators to implement family meetings as part of planned nursing process. Education in a more family-oriented nursing approach seems essential to increase nurses’ awareness with regard to the importance of family caregivers of older patients in the hospital. Implications for education could be to reflect these study findings with nurses in a group discussion to enhance nurses’ awareness of the importance of family caregivers. In most western societies, it is a trend that professional care at home is shifting to care that is less formal and primarily performed by family caregivers. The results of this study might, therefore, be predominantly relevant for health care professionals in western societies. Nevertheless, it is likely that similar results might be applicable in other countries; therefore, reproduction of this study in other countries could be relevant using this study as a reference. Causes of why nurses do not elaborate on aspects of family caregiving could be an important aim for further research.

## Additional file

Additional file 1: Age; admission reason; type and number of chronic conditions and living situation of patients and presence of family caregiver during admission interviews, family meeting and discharge interviews. (PDF 212 kb)

## Abbreviations

CABG: Coronary artery bypass grafting
COPD: Chronic obstructive pulmonary disease
CVA: Cardiovascular accident
F: Female
FC: Family caregiver
M: Male
MD: Missing data
MEC: Medical ethical committee
NA: Not applicable
No: Number
SD: Standard deviation
TIA: Transient ischemic attack
WMO: Wet Medisch Wetenschappelijk Onderzoek (Medical Scientific Research law)
