# Supplementary material for: Aspects of family caregiving as addressed in planned discussions between nurses, patients with chronic diseases and family caregivers: a qualitative content analysis
Source: BMC Nurs. 2017 Jul 11;16:37. doi: 10.1186/s12912-017-0231-5 (PMC5504762; doi:10.1186/s12912-017-0231-5)
Supplement: Additional file 1: — Age; admission reason; type and number of chronic conditions and living situation of patients and presence of family caregiver during admission interviews, family meeting and discharge interviews. (PDF 212 kb) [file 12912_2017_231_MOESM1_ESM.pdf]

Supplementary information on patients and on presence of family caregivers during planned discussions

No code	sex	admission reason	type of chronic condition	living situation	FC present at admission interview	FC present at family meeting	FC present at discharge interview
1 A1	m	exacerbation COPD	pulmonary (2) + cardiovasc. disease + cancer	with partner	spouse	NA	spouse
2 A2	f	exacerbation COPD	pulmonary disease + rheumatism	with partner	none present	none present	spouse
3 A3	m	exacerbation COPD	pulmonary disease (2) + blood pressure with partner issues		none present	NA	none present
4 A4	m	set up coagulation meds	diabetes + cancer cardiovasc. disease	alone	daughter	md	none present
5 A5	m	pneumonia	pulmonary + cardiovasc. disease + diabetes with partner		spouse	none present	spouse
6 A6	f	exacerbation asthma	pulmonary disease (2) + blood pressure issues	alone	daughter + son	NA	daughter + man
7 A7	f	optimizing lung function	pulmonary disease	with partner	spouse	NA	none present
8 A8	m	COPD; optimizing lung function	pulmonary disease + bowel disorder + blood pressure issues	with partner	spouse	NA	none present
9 A9	f	anaemia /abdominal discomfort	cardiovasc. disease + anaemia + thyroid problems	with partner	spouse + daughter in law	spouse + daughter + son in law	spouse + daughter in law
10 A10	m	urinary tract infection/ possible delirium	cardiovasc. disease (2) + blood pressure issues+ kidney disease	with partner	spouse + son	spouse + 2 sons	spouse + son
11 B1	m	pacemaker implantation	cardiovasc. disease (3)	with partner	none present	NA	none present
12 B2	f	congestive heart failure	cardiovasc. disease	alone	son + woman	NA	son +woman

No code	sex	admission reason	type of chronic condition	living situation	FC present at admission interview	FC present at family meeting	FC present at discharge interview
13 B3	f	congestive heart failure	cardiovasc. disease	with partner	spouse + son	NA	None present
14 B4	m	collaps in gym	cardiovasc. disease (2) + diabetes	with partner	none present	NA	spouse
15 B5	m	rhythm observation	cardiovasc. disease + diabetes	with partner	none present	NA	spouse + woman + man
16 B6	m	fluid retention due to reduced pump function	cardiovasc. disease	with partner	spouse	NA	man
17 B7	f	congestive heart failure	cardiovasc. disease + diabetes + stroke	with partner	none present	NA	neighbour
18 B8	m	congestive heart failure	pulmonary + cardiovasc. disease + diabetes	with partner	spouse + 1 man	spouse + son	spouse + 1 man
19 B9	f	cardiovascular accident	pulmonary disease + stroke	with partner	daughter	daughter	daughter
20 B10	f	cardiovascular accident	stroke + cardiovasc. disease	alone	daughter + son	3 daughters + 1 son	2 daughters
21 B11	f	gait disturbance	arthrosis	alone	2 sons + 1 daughter in law	(1): son. (2): son + daughter in law	2 daughters
22 B12	f	cardiovascular accident	stroke + thyroid problem	alone	son in law	son in law	partner
23 B13	m	pain neck / shoulder blades	cardiovasc. disease + hypercholesterolemia blood pressure issues+	with partner	spouse + daughter	spouse + daughter	spouse
24 B14	m	cardiovascular accident	kidney disease + cardiovasc. disease + blood pressure issues	with partner	spouse + daughter	spouse + daughter	spouse + daughter
25 B15	f	insult	pulmonary + cardiovasc. disease	with partner	spouse + daughter + man	spouse	spouse

No code	sex	admission reason	type of chronic condition	living situation	FC present at admission interview	FC present at family meeting	FC present at discharge interview
26 B16	m	lung surgery	oesophageal disorder + pulmonary disease	with partner	spouse + daughter	NA	none present
27 B17	m	suspected mesothelioma	cardiovasc. disease	with partner	woman	NA	daughter
28 B18	m	hypertension	blood pressure issues	with partner	none present	spouse	spouse
29 B19	f	stomach / intestinal complaints	diabetes + cardiovasc. disease	with daughter	daughter	NA	daughter
30 B20	f	pulmonary embolism	diabetes	with partner	spouse	spouse	spouse
31 B21	m	diarrhea and vomiting	diabetes + parkinsonism	with partner	Spouse + son	NA	none present
32 B22	m	possible pneumonia	blood pressure issues+ stroke + pulmonary disease	with partner	spouse + son	md	spouse + son
33 B23	m	hypercalcemia/ liver metastases	diabetes + cardiovasc. disease	with partner	spouse	md	spouse + son
34 C1	f	rehabilitation CABG	pulmonary disease + hypercholesterolemia	alone	none present	md	1 woman
35 C2	m	congestive heart failure	diabetes + cardiovascular + pulmonary disease	alone	none present	NA	none present
36 C3	m	exacerbation COPD	pulmonary + cardiovasc. disease (2)	with partner	none present	NA	none present
37 C4	m	chest pain	hypercholesterolemia + kidney disease	with partner	spouse	NA	none present
38 C5	m	pleural fluid left thorax	rheumatism + diabetes + cancer	with partner	none present	spouse + daughter+ son+ daughter in law	spouse + daughter + son+ daughter in law
39 C6	f	exacerbation COPD	pulmonary disease + rheumatism	with partner	none present	NA	none present

No code	sex	admission reason	type of chronic condition	living situation	FC present at admission interview	FC present at family meeting	FC present at discharge interview
40 C7	m	insult /hip fracture	stroke	alone	son+1 woman	md	son
41 C8	f	clinical lymphedema program	cancer	alone	spouse + son	NA	none present
42 C9	m	cardiovascular accident	stroke	with partner	woman	NA	2 women
43 C10	f	possible CVA	stroke	with partner	spouse + daughter +son	NA	spouse
44 C11	f	meningioma	thyroid problem	with partner	spouse + daughter + man	spouse + daughter man	spouse
45 C12	m	insult	blood pressure issues	with partner	spouse	NA	none present
46 C13	f	fluid retention due to prednisone	diabetes + rheumatism	with partner + daughter	daughter	NA	daughter
47 C14	f	anaemia / kidney insufficiency	blood pressure issues	alone	daughter	daughter + daughter in law	daughter
48 C15	f	nausea	pulmonary disease + diabetes + kidney disease	alone	2 daughters	2 daughters	2 daughters
49 C16	f	diagnostic research	diabetes	with son	son	NA	son
50 D1	f	pneumonia	pulmonary disease + blood pressure issues	alone	spouse	none present	spouse
51 D2	m	dyspnea with pneumonia	blood pressure issues+ rheumatism	with partner	spouse + son	md	spouse
52 D3	m	viral respiratory infection / fluid retention	pulmonary + cardiovasc. disease + parkinsonism	with partner	spouse	NA	spouse

No code	sex	admission reason	type of chronic condition	living situation	FC present at admission interview	FC present at family meeting	FC present at discharge interview
53 D4	f	osteoporosis	osteoporosis + stroke	alone	daughter	NA	none present
54 D5	f	possible CVA	arthrosis + parkinsonism	alone	none present	son + friend+ 1 man+1 woman	friend
55 D6	f	trauma capitis	diabetes	alone	daughter	none present	woman
56 D7	f	acute headache	diabetes	alone	none present	NA	none present
57 D8	m	suspected TIA	blood pressure issues+ hypercholesterolemia	with partner	none present	NA	none present
58 D9	m	dysregulated diabetes	pulmonary + cardiovasc. disease + diabetes	with partner	spouse	NA	none present
59 D10	m	rectal haemorrhage	pulmonary disease + diabetes	with partner	spouse	NA	spouse
60 D11	f	digestive tract bleeding	pulmonary + cardiovasc. disease + arthrosis	alone	son	NA	none present
61 D12	m	rectal haemorrhage	bowel disorder	in living community	neighbour	NA	woman+ child
62 D13	f	dysregulated diabetes	diabetes	alone	daughter in law	NA	none present

CABG: coronary artery bypass grafting COPD: chronic obstructive pulmonary disease; CVA: cardiovascular accident; f: female; FC: Family Caregiver; m: male; MD: missing data; NA: not applicable; No: number; TIA: transient ischemic attack
